# On the Relations between Dental Lesions and Diseases of the Eye

**Published:** 1884-04

**Authors:** Henry Power

**Affiliations:** Ophthalmic Surgeon at Bartholomew's Hospital, and Surgeon to the Royal Westminister Ophthalmic Hospital, &c.


AMERICAN JOURNAL

OF

DENTAL SCIENCE.

VOL. XVII. THIRD SERIES—APRIL, 1884. No. 12.

ARTICLE I.

### ON THE RELATIONS BETWEEN DENTAL LESIONS AND DISEASES OF THE EYE.

BY HENRY POWER, M. B. LOND., F. R. C. S.

(Ophthalmic Surgeon at Bartholomew's Hospital, and Surgeon to the Royal Westminister Ophthalmic Hospital, &c.)

The connection between dental diseases and affections of the eye is not at first sight very apparent, but it has been noticed, though for the most part only casually, by many writers. I have thought that it might interest this Society, and might at the same time be serviceable to those who are engaged in ophthalmic practice, if I attempted to collect some of the evidence which seems to demonstrate that dental disease may excite various ophthalmic affections; that it might also be the means of eliciting valuable hints from those who are engaged in dental surgery, and that it might afford an opportunity for the citation of cases which, though exceedingly interesting when considered in connection with others of the same nature, may not have been regarded by those in whose experience they have occurred as of sufficient importance to merit reporting.

I have taken some trouble in looking through the treatises of those authors who have written in the course of the last hundred years, and I thtnk it may be said that the connection between Dental and Ophthalmic diseases has not been specially dwelt upon in this country before the year 1824. Beer, indeed, before this spoke of a consensual affection of the eye which might occasionally be due to the carious back teeth ; but no mention is made of the teeth by Ware or by Chandler, who both wrote in 1780, nor by Scarpa, whose treatise was translated by Briggs in 1806, nor by Weller in 1821. Scarpa, indeed, in his account of ptosis, states that it may be produced by indigestion, and gastric disease, and by the presence of worms ; and in speaking of amaurosis holds that it may result from contusion and laceration of the supra-orbital nerve.* Weller (1821), in like manner, who copied the great German oculist Beer, though he does not allude to the teeth, andof course does not speak of reflex actions, yet recognizes this class of affections when he refers various forms of amaurosis to sudden suppression of lactation and acute cutaneous affections, and to loaded bowels, whilst he elsewhere refers strabismus and blepharospasm to worms in the intestinal canal. The first writers I have met with who distinctly refer to the teeth are Travers, who speaks of strabismus caused by difficult dentition, and Frick, who, in 1826, speaking of the causes of stabismus, remarks that it is "often concomitant with difficult dentition;" whilst Welbank, who translated Frick's book, in a note on the treatment of amaurosis, states that he has obtained good results from the employment of carbonate of iron in cases of amaurosis "proceeding from disease of the dental nerves."

After this period, difficult dentition and dental disease are often mentioned incidentally amongst other causes of ophthalmic disease.

I presume that you will all accept as a fact, that which is admitted by all ophthalmic surgeons, that there is such an affection as reflex or sympathetic ophthalmia, that is to say, that irritation of one set of branches of the fifth will affect

*Scarpa (p. 511) gives many cases of reflex amaurosis.

those of the opposite side; yet in discussing the subject before us it seems to me in the first place important that I should very briefly put before you the evidence showing that reflex irritation of the eye really occurs. If any ophthalmic surgeon be asked why he believes in reflex irritation of the globe and its appendages, his mind at once reverts to many cases in which injury to one eye, especially of the ciliary region, has been followed, after a period of very variable duration, by inflammation of the uninjured eye of a low but steadily progressive type, generally involving all the tissues, and leading to more or less serious impairment, or even to complete loss, of vision.

I could give many cases from my own experience. I will limit myself to two.

A young man presented himself to me at St. Bartholomew's Hospital, Chatham, saying he had been struck with a piece of the head of a chisel on the eye. His vision had previously been perfect. On examination there was a wound in the cornea, a rent in the iris, blood in the anterior chamber, and traumatic cataract. He could see light, and the shadow of the hand. I had no doubt that a fragment of metal was in his eye, and that practically the eye was lost. I recommended its removal. He declined. A week after, perception of light still remaining, he again declined to have anything done. Soothing remedies were employed. About six weeks' later he came with the opposite eye sharply inflamed: there was dimness of vision and general inflammation of the globe. He was now anxious to have the eye originally damaged, from which he had suffered a martyrdom, removed. I did that operation, but it was too late; the inflammation ran its course in spite of careful treatment, and he lost the sight of both eyes. That is the usual result.

A second case. Two lads sitting at the same desk. One presents a steel pen to the eye of the other, when his head is averted, and calls his name; he turns his head to the speaker, and the nib of the pen makes a punctured wound at the outer border of the cornea. A slight protrusion of the iris takes place. He continues his work, with gradually increasing irritation in the damaged eye, for three weeks, when suddenly vision in the second eye commences to be impaired. He is unable to accommodate his eye for near objects, and iritis sets in. The gravest anxiety is felt that total loss of vision will ensue, but fortunately this result was averted; by keeping the lad in perfect darkness for a month, and by appropriate local and general treatment, the reflex or sympathetic irritation gradually subsided, and by a singular piece of good fortune the injured eye also quieted down, and absolutely perfect vision was recovered in both eyes. This is an unusual result of sympathetic ophthalmia.

It is, however, by no means necessary that irritation, even when it is very severe on one side, should necessarily radiate to the opposite eye, or to other nerves.

Curious cases sometimes occur, and it is as difficult to say why inflammation does not arise in one case as it is why it does not occur in another.

About 1870 a man presented himself to me at the same clinic with severe injury of the eye, also from a piece of iron broken off the head of a rivet: the eye was laid open by the fragment, and vision was lost. I recommended removal of the globe. The man declined. He went to Maidstone. The surgeon—I believe, Mr. Adams—gave him, he said, the same advice, but he was determined not to have it done. He recommenced his work, and the eye gradually quieted down, but from time to time he suffered severe pain in it, and had to discontinue work for some days; still the other eye remained strong. About two months ago, however he called at the hospital, and then had conjunctivitis and some impairment of vision in the healthy eye, whilst the opposite one was tender and painful. I exhorted him to have it removed. He reluctantly, and only after a week or two of deliberation, consented. In the operation for removal I met with a difficulty. After dividing the conjunctiva and muscles, I introduced a pair of scissors, but was unable to divide the optic nerve. Larger and longer scissors was tried, and it was only after considerable trouble that the globe was enucleated. The reason of the difficulty immediately appeared, for a large piece of metal was embedded in the opic nerve, just where it might be expected that it would excite sympathetic ophthalmia, yet where it had remained for twelve years, with but slight effect on the opposite eye; for he made a good recovery, and was discharged on the 17th October, 1883, and has had no trouble since.

If the question be put, how does the irritation or inflammation of one eye come to effect the other—by what path, or paths, does the irritation travel? the reply given, both by exact clinical observation and by pathological investigation, is that in the vast majority of cases there is a neuritis which travels along the ciliary nerves, but in a few instances along the optic nerves, and we shall see that this conduction of the morbid process along the sheaths of the nerve is of considerable importance in enabling some explanation to be given of the occurrence of reflex troubles where the teeth are the seat of the primary lesion.

Injuries affecting the branches of the first division of the fifth pair are well known to affect the eye of the same side, and there are good reasons also for believing that affections of other branches of the fifth pair of nerves may be the cause of ophthalmic troubles.

In 1853, M. Decaisne, staff surgeon of the Belgian army, presented to the Academie de Belgique,* under the title of "Sur les dents œillıeres," a note on the disturbances of vision consequent on alterations of the teeth, in which he states that he saw an officer struck on the forehead with a piece of wood in the course of the frontal nerve, which caused complete blindness, and then remarks that it is not uncommon to see diplopia, or even complete loss of vision, supervene, either with or without ulceration of the cornea, as a result of disease of the maxillary sinus.

---

* "Bull de l'Acad. Med. Belg.," T. XIII., 1853, and "Archiv. Med. Mil.," 1854.

That injuries to the supra-orbital nerve are occasionally followed by amaurosis seems to be well established. A case is recorded by Valsalva* of similar damage in which recovery is stated to have taken place. Unfortunately, however, in these cases the older authors had no means of knowing whether the deeper structures of the eye were injured or not. Some of the cases recorded might well have been accompanied by dislocation of the lens, by hæmorrhage in or detachment of the retina, by crushing of the optic nerve in the optic foramen, and by other conditions which the invention of the ophthalmoscope would now enable us readily to diagnose, and which can in no way be regarded as of a reflex nature. In one case, for example, the patient was thrown out of a wagon, and in another severely pecked by a turkey-cock. Severe blows and cuts over the brow and cheek may easily produce blindness by intra-ocular mischief, without causing any apparent damage to the surface of the eye. Thus Nossi † reports a case where a peasant was struck by a knife which cut the brow. M. Nicaise ‡ states that he has on various occasions noticed the coincidence that exists between affections of the nasal fossæ and those of the eye. He believes he has thus been able to recognize a connection between the occurrence of nasal polypi, and glaucoma, attributable to irritation of the many branches of the fifth pair which are distributed to the mucous membrane lining the nasal cavity. Demours § gives a case of amaurosis supervening after extirpation, *secundum artem*, of a sebaceous cyst of the size of a hazel-nut; the same evening inflammation of the eye supervened, and the next morning the eye was lost.

In 1870, M. Delestre published an essay on accidents following extraction of the teeth, and reports several cases which tend to prove the existence of visual troubles. He remarks that odontalgia is often accompanied with weeping

* "Dissert.," II and XI.

† Himly, i., 89

‡ "Gaz. Med. be Paris," 1871, p. 150.

§ T. I., p. 173.

and redness of the conjunctiva, with sudden darts of pain, and winking of the lids. He considers that there is at first irritation of the branch of the dentary nerve supplying the affected tooth, and which is therefore one of the branches of the superior or of the inferior maxillary nerve. This irritation of one part of the fifth extends to other parts of the fifth, and particularly to the ophthalmic, which leads to lachrymation, and redness of the conjunctiva.

That the teeth and the eye may be concomitantly affected, as the result of some widespread and general disease of the system, as in syphilis, Mr. Hutchinson's observations have made abundantly clear, though there is little doubt that some cases closely resembling those which he has so well and carefully described are the result of other conditions of the system than those appertaining to syphilis; at all events, they occur in children in whom no evidence of syphilis can be obtained from either parent.

I apprehend, however, that this is not exactly included in the subject on which I am about to speak to-night. My object is rather to bring before you some evidence of the occurrence of ophthalmic affections as a consequence of dental disease. And the grand difficulty that I have met with in drawing any definite conclusions, is the extreme frequency with which dental affections present themselves. Thus, to take one affection alone, I was desirous of ascertaining whether dental caries and phlyctenular ulcer of the cornea stood to each other in the relation of cause to effect; but on examining the teeth of my young patients with phlyctenulæ, I found they all had carious teeth. It might be said, is not that evidence that there is a close relation between the two affections? but, unfortunately for this view, the disease can readily be cured—in many instances—by the means usually adopted in ophthalmic practice without the extraction of the offending teeth, so that they only fulfil half the old adage constituting *a vera causa*, "*præsens morbum facit, sublata tollit.*"

Having, however, established the existence of reflex irritation of the eye, it will perhaps be the best mode of treating of the various modes in which that irritation may express itself, if we consider it under the three following heads:—

1. Reflex irritation affecting striated and unstriated muscle.

2. Reflex irritation affecting the mucous membrane and cornea.

3. Reflex irritation affecting the optic nerve and retina› and intraocular tissues.

In regard to reflex irritation affecting muscular tissues, we may mention—

1. Paresis of the ciliary muscle.
2. Paresis of the intra-orbital muscle.
3. Paresis of the muscular fibres of the iris.
4. Paresis of one of the ocular muscles.
5. Paresis of the orbicularis palpberarum muscle.

One of the commonest forms of visual disturbance, induced by dental disease, is *loss or failure of the power of accommodation*. By power of accommodation is meant the faculty which the eye possesses of adjusting its refraction so that well defined images of objects situated at different distances from the eye may be formed upon the retina. This is accomplished, as is now well known, by the action of the ciliary muscle, which, by contracting, causes the lens to become thicker. For the lens is normally and, when the eye is at rest, covered anteriorly, and flattened by the anterior capsule, which is stretched by the suspensory ligament; but when near objects are fixed, the ciliary muscle is brought into play. This brings forward the anterior part of the choroid, and relapses the suspensory ligament, and the lens immediately, by virtue of its own elasticity, becomes thicker, and is thus rendered capable of focussing divergent rays —that is to say, rays proceeding from near objects. The stronger the muscle, and the more elastic the lens, the greater is the degree of accommodation which can be effected; for, under these circumstances, the suspensory ligament can be thoroughly relaxed, and the lens can become highly convex. Hence accommodation is strong, or has a great range in children, extending from infinite distance to a point not more than three or four inches from the eye ; for in them the ciliary muscle has great power, and the lens is very elastic. In advanced age, on the contrary, the muscle becomes feebler, and the lens is of firmer consistence and much less elastic. The suspensory ligament, consequently, does not undergo much relaxation, and the lens does not swell when the compressing influence of the anterior capsule is withdrawn. Hence the range of accommodation diminishes : distant objects, from which parallel, or approximately parallel, rays proceed, are still accurately focussed upon the retina ; but any small object, to be clearly seen, must be held at a distance of 10 to 12 or 18 inches from the eye.

The influence of dental lesions on the accommodation has been particularly investigated by Dr. Hermann Schmidt, of Berlin. This observer examined the eyes of ninety-two patients, who presented themselves at his clinic, suffering from some form of dental disease—such, for example, as caries, periostitis, or neuralgia. Considerable pains were taken to determine the range of accommodation in both eyes, and to compare that on the sound with that on the unsound side ; whilst reference was made to the tables of averages for each eye given by Donders. Amongst the ninety-two cases, Schmidt found there were only nineteen cases in which the range of accommodation was normal, or better than normal ; in the remaining seventy-three cases the range was lowered, and in most instances considerably reduced.

In unilateral odontalgia, the range of accommodation was reduced on the same side in thirty cases. In fifty-one cases there was no difference in the diminution of the range of accommodation perceptible. In one case, the eye of the side opposite the unsound teeth was the weaker of the two. In nine cases the teeth were carious on both sides. It appeared further that the influence of dental lesions on the accommodation was more frequent the younger the patient ; the ages between ten and twenty being those in which the paresis was most marked. After thirty years of age the impairment of the power of accommodation was much less frequently observed.

The influence of sex was not noticeable.

In regard to the locality of the dental lesion; in forty-one cases in which the upper maxilla was affected, paresis of accommodation was observed seventeen times. In thirty-nine cases in which the lower jaw was affected, the paresis was observed nineteen times. Disease of the teeth of the lower jaw, therefore, appeared to be somewhat more effective in producing failure of accommodation than that of the upper jaw. Disease of the incisors, and of the first, third and fifth back teeth, using the German mode of notation, appeared to be more frequently associated with the ocular disturbance than lesion of the canines, or of the second and fourth back teeth.

The nature of the dental lesion appeared to be of little importance, great impairment of accommodation accompanying comparatively slight disease, whilst extensive disease of the teeth, accompanied by severe pain, was associated in some instances with only slight paresis of accommodation.

Dental lesions appeared to have no effect upon the sharpness of vision.

The impairment of the power of accommodation was only rarely noticed by the patients themselves, which perhaps might be in part attributable to the circumstance that in most cases, if the patients were suffering much pain, the ordinary avocations were suspended.

The explanation of this remarkable effect of dental lesion is not quite clear. Two views may, it appears to me, be taken of it. We may regard it as of a reflex character, and as being analogous to that weakness or paralysis of certain muscles observed in some of the reflex paralysis of infancy, and which we may consider to be of the nature of an inhibitory influence, since they rapidly supervene and rapidly vanish; or we may regard it as a simple result of the generally lowered tone of the system, consequent on the depressing influence exerted by the dental lesion. I need hardly dwell in this place, and before such an audience, on the extraordinary powerful effect on the nervo-muscular and circulatory systems of prolonged toothache, or how the impairment of the digestive functions, the sleeplessness, and general feeling of malaise, lower the tone of the nervous system, and produce indisposition for all kinds of mental and bodily exertion. There can be little doubt that the sympathetic nervous system and the unstriared muscles participate in this depression; and we can easily understand how there is a corresponding diminution in the power of the ciliary muscle, and failure of accommodation.

Schmidt himself advances a very different explanation. He believes that in these cases a reflex stimulation of the vaso-motor nerve takes place, leading to increased intra-ocular pressure, and consequently to impairment of the power of accommodation. But when Schmidt's paper was written the action of the vaso-motor nerves was less known than at present. And such reflex stimulation would seem calculated to lead to a diminution, rather than to an increase, of intra-ocular tension.

To enable us to speak quite positively upon the influence of carious or painful teeth upon the accommodation we require information that is very rarely attainable. We ought to know that the accommodation was thoroughly good before the teeth were affected, that the failure of accommodation occurred simultaneously, or soon after the dental lesion; and, finally, that after the removal of teeth, recovery of the accommodation power takes place.

In the majority of cases we must rely upon the patient's word for the first of these factors of our judgment; and, in regard to the second, so many persons of both sexes, and all ages, are affected with dental disease that if this be a *vera causa* good accommodation would be the possession of only a favored few: whilst lastly, even Schmidt, who paid particular attention to the subject, was only able to examine eight cases, out of nearly a hundred, after the extraction of the offending tooth or teeth. In five of these, however, distinct improvement was observed. The diminution in the power of accommodation was in each case characterized by the recession of the near point, whilst the far point remained unaltered. After extraction of the affected teeth the near point approximated to the eye.

Another class of cases in which a reflex influence appears to be exerted upon muscular tissue is that in which the muscular tissue of the iris is affected. This condition is termed *iridoplegia* or *mydriasis.* In it the pupil is widely dilated and motionless, owing, as is generally believed, to paralysis of the spincter pupillæ, which is supplied by the ciliary nerves containing branches of the third nerve; but it is possible that it may be due to contraction of the dilatator iridis, if such a muscle really exists, and this might be produced either by irritation of the branches of the sympathetic or of the fifth pair, which Bilogh believes supply it.*

It is remarkable and suggestive of its mode of origin that mydriasis rarely if ever occurs simultaneously on both sides, and in many cases that I have seen in past years I have scarcely ever been able to trace the ætiology, though I must admit that I have not paid special attention to the teeth. It is instructive to find that in one case recorded by Desmarres, a cure was effected by the extraction of a carious molar, and in future I shall certainly in any case of the kind that may present itself, make a careful examination of the teeth.

In the cases that have fallen under my care the dilatation of the pupil has been permanent, which points rather to some organic lesion, such as might be produced by neuritis, than to any inhibitory influence.

One case, and so far as I know one case only, of *exophthalmia* has been placed on record. It is contained in a recent number of the "*Recueil d'Ophthalmologie*" of Galezowski, 1882, p. 441, and is reported by Weinberg. The chief facts of the case are that a widow presented herself in the out-patient department with an exophthalmia, accom-

* Wecker, i., 407.

panied by severe pain of the right eye. She had suffered for thirty-five years from epileptic vertigo and cramp of the stomach. In the month of March last year she had an attack of phlebitis of the left femoral vein, and on examination she had varices in this leg, and some œdema of the left leg. For five weeks previously she had experienced noises in the right ear, with fronto-temporal cephalagia normal aspect, and she had intermittent sleeplessness. The pricking sensations gave place to lancinating pain, the eye reddened, and the bulbar conjunctiva was strongly injected. For four days previous to her appearance the vision had become hazy.

On examination the tension was found to be + 1, and there was exophthalmia of the right eye. The cornea was hazy; the palpebral and bulbar conjunctiva were much injected; the movements of the eye outwards and inwards were insufficient. Ophthalmoscopic examination showed floating particles in the vitreous, and as she was *myopic*, a cresent; the peripheric retinal veins were injected. The whole retina was redder than natural; near the macula were several hæmorrhage spots. The heart, arteries, lungs, and the urine were normal. She could read No. 1 at 3 inches distance. Two leeches were ordered for the right temple; alternating instillations of atropine and eserine were ordered, but the former gave pain and was discontinued.

Ten days later, severe periorbital pain being experienced, with acute chemosis, the teeth were examined and two carious teeth and a stump were found and promptly extracted. The results were remarkable. In the course of three days, the patient was greatly improved in health, and the chemosis had disappeared, as well as the injection of the conjunctiva, and above all the exophthalmia.

In such a case as this we might attribute the relaxation of the vessels spoken of as conjunctivitis to paralysis of the vaso-motor system, and consequent passive dilatation of the conjunctival capillaries; whilst the protrusion of the globe may have been due to relaxation and loss of tone of

Turner's periorbital unstriated muscle and of the several recti muscles, as well as to relaxation of the vascular and lymphatic systems behind the globe—all conditions that are explicable on an inhibitory influence being exerted by the damaged tooth.

That *strabismus* may be induced by dentition, and especially by difficult dentition, is, I think, generally admitted. Travers, as long ago as 1824, wrote: "Strabismus is also a symptom of irritation arising from difficult dentition." Many cases of strabismus come on quite suddenly, sometimes without the occurrence of any fit, but oftentimes immediately after a fit, during the first year of life, and therefore when the muscular exertion of the eye, requisite to be made to effect accommodation can have had nothing to do with it; for few children look intently at near objects, when alone the accommodation is brought into play, during the first few months of their existence. Yet the opinion that dentition is a cause of strabismus seems to rest on general consensus of opinion only, rather than upon any special well authenticated cases. And the frequency with which it is referred to in recent books is much less than was formerly customary; in fact, in some of the more recent treatises in Germany and France it is not even mentioned. This is attributable to the important generalisation of the ætiology of strabismus made by Donders, that in by far the greater number of cases it is due to hypermetropia.

The time at my disposal will not permit me to enter into any details in regard to the mode in which hypermetropia, or long-sightedness, leads to stabismus, but it will be enough to say that the hypermetropic eye is a flattened eye, that in accommodation for near objects great convergence of the eyes is required, and that a powerful and sustained nervous impulse is required in order that a near object should be fixed by both eyes, and that, owing to defective association between the effort for accommodation and that for convergence, convergent squint is frequently induced.

Other striated muscles that have been noticed as being occasionally affected in a reflex manner by disease of the teeth have been the levator palpebræ and the orbicularis palpebrarum ; the former supplied by the third nerve, the latter by the seventh ; the paralysis or paresis of the former leading to more or less expressed *ptosis*, the latter to *lagophthalmos*. Ely* has recorded three good cases of such affections, in one of which there was paresis of the orbicularis palpebrarum, with irregular spasm of the ciliary muscle and monocular diplopia. In another there was paresis of the rectus internus and ciliary muscles, and in a third case partial paresis of the third nerve.

An affection that is not unfrequently observed is that which is termed *blepharospasm* or incessant winking of the lids, the movement being sometimes limited to the orbicularis palpebrarum, but at others extending to other muscles, such as the zygomatici and levatores anguli oris. This affection is well known to be associated with the error of refraction known as hypermetrópia, when a powerful effort of contraction of the ciliary muscle is demanded. The impulse emanating from the brain, instead of being limited to the nerve supplying the ciliary muscle, has a strong tendency to radiate through other channels, and consequently to affect other muscles ; whilst in some instances the secreto-motory fibres of the fifth nerve are excited, and a copious discharge of tears results.

But that similar conditions may be established in a reflex manner by some lesion of other nerves is well shown by a case that was reported many years ago, by Von Grafe, to the Medical Society of Berlin. In this instance the blepharospasm ceased almost instantaneously when pressure was made upon a point situated below the alveolus of the third inferior molar tooth. At this point an incision was made down to the bone, but without modifying the blepharospasm in any appreciable degree. But having then established the fact that compression of the sub-orbital nerve and of the temporal branch of the molar materially diminished the spasm, section of these nerves was performed.

---

* "New York Med. Record," Nov. 11th, 1882.

The spasm immediately ceased, and the case was thought to be cured, but it re-appeared at the expiration of a fortnight. The section of the inferior dental nerve performed within the mouth ultimately caused the blepharospasm to cease entirely, and it had not re-appeared four weeks after. A case of convulsive tic, as he terms it, has also been recorded by Mitchell, which, supervening spontaneously, was propagated to the muscles of the neck and arms, and which entirely disappeared after the removal of several teeth.

As additional evidence of the influence cf the stimulation of other and distant parts of the nervous system on the orbicularis may be mentioned the observations of Claude Bernard; for this experimenter found that section of the sympathetic is followed by partial closure of the lids, and inversely if blepharospasm is induced in an animal by irritating the cornea with some caustic substance it may be removed by galvanizing the sympathetic in the neck, when the lids open immediately, as by magic.

From the consideration of the influence of carious and painful teeth upon the striated and unstriated muscular tissue of the eye, I pass to that which they may be supposed to exert upon the conjunctiva, cornea, and sclerotic. And I would refer, in the first instance, to the affection named phlyctenular ophthalmia, which is very frequently associated with carious teeth, and which I believe to be often caused by them.

*Phlyctenular ophthalmia* consists in the appearance of one or more small vesicles, soon bursting and forming ulcers around the margin of the cornea, or on the surface of the cornea, or of the sclerotic. Sometimes only one is formed; at others a succession appears. They sometimes produce little or no uneasiness; at others they are attended with great intolerance of light, and abundant lachrymation. The child—for the disease occurs more frequently in children—keeps in the dark during the day, and only becomes lively at night.

The pathology of this disease is tolerably well known. It is believed to result from an inflammatory process, attended with the proliferation of nuclei along the sheaths of the nerve. The nuclei gradually advance towards the surface along the conjunctival, corneal, and sclerotic branches of the fifth nerve, and accumulating on the surface, form, with a little fluid, the contents of the vesicle, and when the vesicle bursts they gradually break down and disappear.

Now in cases of phlyctenular disease I have been accustomed to give minute directions in regard to diet, believing that this affection owed its origin to reflex irritation of the nerves of the eye, consequent upon some disturbance of the stomach and intestinal canal; and to the mothers of the numerous little patients suffering from this affection in our ophthalmic hospitals and departments of hospitals, I have been accustomed, after ordering a brisk purge and some quinine, to say, "attend to its diet; no sweets, no pastry, no raw fruit. Give it bread and milk, finely cut-up meat and potatoes," and I am perfectly satisfied with the result of that treatment; but it has occurred to me that the view I have hitherto entertained of the ætiology of the disease may be erroneous, and that instead of the stomach, the teeth are perhaps the organs at fault. It is easy to comprehend that the mastication of raw apples, candied lemon and orange peel, jam tarts and the like, lead to the impaction of acid and fermentable substances in the cracks and crannies of the teeth, especially if these are carious, and that remaining there for hours or days, if the due cleansing of the mouth after food is neglected, such particles may set up inflammatory troubles which may propagate themselves along the nerve and lead to what we term reflex phlyctenular inflammation of the eye.

No doubt there are many exceptions to the rule that carious teeth and phlyctenula of the conjunctiva are associated, but I have noticed that a large proportion of the children who have phlyctenular ophthalmia have also carious teeth. Surely this is very suggestive of the ætiology of the affection.

The occurrence of inflammation of the conjunctiva in such cases as the foregoing, as Weinberg remarks,* may be explained on two theories: on the one hand, when any organ is under the influence of some stimulus, this influence may modify the nerve filaments which terminate in this organ, and thus occasion phenomena of a reflex nature, either in the eye or elsewhere.

But it may also happen that the irritative process itself, whatever that may be, may propagate itself until it reaches the filaments which excite the neighboring organ. It is evident that this can only happen when the filaments supplying the affected organs are derived from the same nerve, or when there are anastomoses between two nerves distributed to different organs. The two kinds of propagation, he observes, are well exemplified in the case of a carious tooth. In one instance there shall be well-marked conjunctivitis which resists ordinary treatment. The mouth is examined and a carious tooth is found, the very existence of which is unknown or forgotton by the patient, so slight has been the inconvenience he has experienced from its presence. The tooth is extracted, and the conjunctivitis forthwith and spontaneously disappears. This is a case of purely reflex action. But other cases are met with in which odontalgia is present, and in which the redness of the conjunctiva is accompanied with the small phlyctenular ulcers to which attention has just been drawn, with chemosis, and some impairment of vision. The pathogeny of this affection is more complex: part of the symptoms are here due to reflex irritation, but part also to an extension of the dental neuretis of the ophthalmic branch.

In the course of nine months' out-patient practice, Weinberg believes he met with 188 cases of ocular disease dependent on dental lesion.

I have some reason for believing that serious lesions of the cornea, may be primarily due to carious teeth, and I will venture to give you the heads of a case that I watched

* "Recueil d'Ophthalmologie," Nov. 1882

with much interest, but which ended disastrously so far as the eye was concerned.

J. F., a stout, ruddy Scotch girl, aged twenty-four, previously healthy and with good family history, suddenly, after exposure to cold, was seized with a sudden sharp pain on left side of face. The vision was then good on that side, but after a few days became dim. A small circumscribed collection of pus formed exactly in the centre of the cornea, and she was then brought to me. She was treated for a few days with quinine and hydrargyrum c. creata, and with atropine drops, no improvement resulting. I performed paracentesis corneæ at the side of the cornea, which the girl bore without flinching. Slight improvement followed. Local depletion, in the form of leeches and blisters, and general tonic remedies, strychnia and iron, were prescribed; but, the case dragging on, I again determined, to tap the cornea, and this time passed the needle through the little abscess. The extreme indifference she showed to the puncture attracted my attention, and I then discovered that the cornea was anæsthetic, and that the sensation of the whole region supplied by the ophthalmic branch of the fifth was greatly impaired, though not quite lost. She was a servant, and as the case was likely to prove a troublesome one, she was taken into St. Bartholomew's Hospital under my care. The abscess had now become an ulcer; the cornea was generally hazy; there was much conjunctivitis, but no chemosis. She could distinguish the points of a pair of compasses at a distance of 1·5 c. m. over right brow, but only at a distance of 4·5 c. m. over left brow. Special attention was then paid to the teeth.

She stated that she had suffered much from toothache, and several carious teeth were found in both jaws, but especially on the left side. Four molars had been extracted at once. In the course of a month from this time the cornea had become clearer; some hyopyon previously present disappeared. The iris could be seen, and vision, which had been almost lost, recovered sufficiently to enable her to count figures, whilst the anæsthesia of the globe almost vanished. She left the hospital and took a cook's place, but she returned about seven months after saying that the eye had slowly deteriorated, perhaps from exposure to heat, and although there was now no loss of sensibility, the inflammation of the cornea and sclerotic, and the general discomfort from the condition of the globe, preventing her from doing her work, was so great that I determined to perform abscission, which was done nearly a year after the commencement of the disease, and the girl made a good recovery.

The lesion of the cornea in such a case as this may be referred to its anæsthetic condition; but the question arises, what caused the anæsthesia? and I can suggest no cause for it except that her teeth were seriously affected.

I now proceed to the consideration of the last class of cases into which I divide the ocular troubles caused by dental lesions, namely, those in which the reflex action is exerted upon the optic nerve and retina and inter-ocular structures.

And first in regard to the occurrence of *amaurosis;* the most brilliant case on record is one that is now of somewhat ancient date, having been given by Sir William Lawrence, but which is so convincing that I venture to reproduce it here.

"Case 7. *Amaurosis Caused by a Carious Tooth.*—F. P., thirty years of age, possessing a good constitution, and enjoying good health, with exception of pains in the head and limbs, which never lasted long, suddenly experienced in the autumn of 1825, a violent pain, shooting from the left temple to the eye and the side of the face: he ascribed it to cold. The pain lasted several days, then lessened, and reappeared from time to time without being sufficiently severe to induce the patient to seek medical aid. In about two months it suddenly increased in intensity, occupying the eye particularly, with a feeling as if it would pass out of the orbit. F. P. now discovered that he was blind with that eye, and applied to a neighboring physician, whose treatment, continued for two months, did no good. The pain, however, was no longer continual: it assumed a somewhat periodical character, leaving the patient easy for some hours of the day. At the end of the following six months the pain increased, the cheek swelled, some spoonfuls of bloody matter were discharged by a spontaneous opening in the lower eyelid, after which the swelling subsided, and the pains nearly disappeared, although the blindness remained complete. The discharge was renewed from time to time during the following six months, and there was no great suffering. But in the autumn and winter (1826) the pain, particularly in the eye, became so violent that F. P. came to Wilna in the beginning of 1827, determined to have the organ extirpated, if no other remedy could be found. Professor Galezowski found the left eye totally insensible to light, with the pupil dilated, and no other visible alteration. The pain, not then so severe, consisted in violent occasional pricking or darting sensations in the left temple and parts round the eye. There was discharge from the lower eyelid. The first molar tooth of the left side was carious; it had not caused much uneasiness; and the toothache, when it existed, had not coincided with the pains in the temple and eye. The Professor determined on removing this tooth, and having done so, was surprised to see a small foreign body at the extremity of the fang. When drawn out, it proved to be a small splinter of wood, about three lines in length, which had traversed the centre of the tooth, and had probably been introduced in picking the teeth. A probe was passed from the socket into the antrum, from which a few drops of thin purulent fluid escaped. The pain ceased almost entirely, and on the same evening the eye began to be sensible to light. Vision gradually improved, so that, on the ninth day, the patient could see as well with the left eye as with the right, after a blindness of thirteen months; on the eleventh day he left Wilna to return to his family."

"I had," says Sir William, "the pleasure of becoming acquainted with Professor Galezowski when he visited England subsequent to this occurrence. He showed me the tooth and the splinter of wood, and he pointed out two circumstances in the case as particularly worthy of notice: first, that the entrance of the foreign body into the tooth had not been noticed at the time; and secondly, that a local irritation hardly perceived at the seat of injury, should have affected the ramifications of the nervous trigeminus so violently as to produce amaurosis."

I met with no other case that is so complete as this; but Caffe* refers to a case in which blindness proceeded from a carious tooth of the upper jaw, and occurred whenever food collected in the cavity; and I have myself recently met with a case in which loss of vision occurred for a few minutes each time that, in clearing the cavity of a carious molar for plugging, the instrument caused pain.

Sir Thomas Watson† refers to the repeated occurrence of blindness cured by the extraction of irregularly arranged teeth. Sudden blindness occurred in a boy eleven years of age. This was immediately cured on the removal of two permanent and four milk molars from a very closely compressed set of teeth; and Garretson ‡ reports a case given by Salter. A woman, twenty-four years of age, suffered from violent pain in the right upper first molar, which was accompanied with great swelling of the face; the globe was protruded, and vision was lost. A fistulous opening, from which pus flowed abundantly, existed at the outer as well as at the inner angle of the antrum. The cause of the suppuration in the antrum was the remains of the first upper molar; when this was removed a sound was passed into the antrum and a sequestrum was brought away. This proved to be the antral part of the floor of the orbit, the upper part of malar bone, with the foramen infra-orbitale, and a broad, bony plate of the nasal wall of the antrum. The inflam-

* "Lancette Francaise," Aug. 22, 1839.

† "London Med. Gazette," fi 5, 1841.

‡ Virchow Hirsch, *loc. cit.*

matory symptoms immediately subsided, but there was no perception of light, and the pupil remained immovable. Five weeks subsequently the pupil began to respond to light, but there was no return of vision. Ophthalmoscopic examination of the eyes showed white atrophy of the optic disks. Gill concludes his essay with the statement that the phenomena which depend upon the optic nerve are attributable to a plastic inflammation, which affects the extra-cranial portion of the nerve. If the optic nerve is destroyed by this means, the blindness is persistent. The oculo-motor nerve suffers temporarily only; therefore also there is only temporary rigidity of the pupil. The protrusion of the bulb indicates that the sixth nerve is little or not at all affected. Mr. Gill is doubtful whether to regard the anæmia of the optic nerve as a cause or a consequence of the abolished function.

A case of sympathetic disease of the eyes in consequence of irritation of the dental nerves is given in the "*Revue de Therap. Med.-Chir.*," Aug. 1871. The ocular and palpebral conjunctivæ of the eyes and eyelids were much congested; the vessels filled to the corneal border. Severe pain was experienced at night; there was considerable photophobia; the head was heavy and painful, tongue coated, appetite bad, pulse small and frequent. The affection lasted five months. Ten leeches were applied to the angle of the jaw, a collyrium of sulphate of zinc and a purgative prescribed, which led to some improvement. After the application of a blister to the back of the neck the pain recommenced As the left molar was very carious it was removed, with the effect of immediately removing the symptoms, and in the course of a week the patient could use the eye again.

Gill, about the year 1873, read a paper before the Missouri State Medical Association, on "Disturbances of Vision Resulting from Neuralgia of the Fifth Pair of Nerves Consequent on Dental Affections," which I have not been able to procure, but which is translated in the "*Deutsches ierteljahrschrift fur Zahnsheitkunde*," B. IV., p. 11, and is fully abstracted in the section on " Mund und Zahnkrankheiten," by Dr. Albrecht, in the "*Jahresbericht fur Gesammten Medicin*," (V. Virchow, V. Hirsch) for 1873, B. H., p. 537. The following case is given in this paper :—The patient was 33 years of age, and in the early part of the winter of 1870–71 began to experience neuralgic pains in the head and face, which lasted to the middle of the summer. The neuralgia extended over both sides of the head, and so far affected the visual power that the patient was no longer capable of reading. In the middle of the summer the neuralgia disappeared, and the vision became normal. In July, 1871, the neuralgia recurred, and coincidently there was diminution of visual power: the patient could only read Jager No. 16. The pain experienced was especially severe at night. Examination with the opthalmoscope showed that there was sub-retinal effusion, and that the retinal arteries were ill-defined. There was a well-marked painful spot.

Examination of the apparently sound teeth showed that there was necrosis of the exposed left superior maxilla, but more careful examination demonstrated that the supposed necrosis was only a collection of tartar, which had surrounded the roots of the first and second molars. These two teeth were extracted, and a wide zone of ulceration was found around their roots, and to a smaller extent about the root of the second bicuspid, which, after the lapse of a few days, was also removed. The neuralgic symptoms immediately abated; the amblyopia, or dimness of vision, was reduced, and in the course of a week the patient could read again No. 6.

Mr. Hutchinson, in one of the early volumes of the Ophthalmic Hospital Reports,* has pointed out that certain cases of amaurosis bear a very close resemblance to infantile paralysis. " Without any warning whatever, without any cerebral symptoms, a patient loses the sight of one eye, and now and then, but very rarely, of both eyes." In some

* Vol. iv., p. 382.

cases, he goes on to remark, there is a certain amount of improvement afterwards, but often the amaurosis becomes total and permanent. In some instances the optic disk becomes white and atrophic, but in others it retains almost normal vascular supply. Most of the cases which had come under his notice were women. In some, evidence of cardiac disease existed; in others there was none; and in most he found the *arteria centralis* of good size, so that the hypothesis of embolism seemed to him to have but little support. In several of the best marked cases that he had seen there was a history of neuralgia in the face for a long time before the occurrence of the amaurosis, the neuralgia being usually connected with the toothache.

Alexander of Aachen has reported* the following case of amaurosis resulting from dental disease:—

A patient, æt. 26, had slight myopic astigmatism, which was, however, insufficient in amount to explain the greatly lowered visual power of the right eye. The right optic disk was somewhat hyperæmic. Abstraction of blood was ineffective, and in a subsequent examination the failure of vision was so great that the patient could only read Jager No. 15. The patient then complained of severe pain in the upper right anterior molar tooth, and observed that it had been for some time periodically affected. The tooth was extracted, and vision almost immediately rose to two-thirds of its normal amount in the right eye, and became quite normal in the left eye.

In this, as well as in other reported cases, the eyes have often been slightly defective originally, and it stands to reason that any sympathetic influence is more likely to be felt by those in whom the functions of the eye are from any other cause impaired; or perhaps it might be said that sympathetic affections are more likely to be super-imposed upon defective than upon sound eyes. Thus a patient who has perfectly healthy eyes may long resist the depressing influence of toothache, but if his eyes are already congested or

* "*Monatsblatter f. Augenheilk*," B. VI., p. 42, 1868.

exhausted from uncorrected hypermetropia, it is easy intelligible that pain, by depressing the nervous energies, will cause the weakness of the ciliary muscle to be felt at an earlier period.

A similar case, in which an amaurotic condition of the eyes was improved by the removal of the stopping from a carious tooth, and was subsequently entirely cured by the removal of the tooth, has been reported by Dr. M. F. de Witt.* A strong and healthy man, æt. thirty-one, discovered that his right eye was blind. No pain, no photopsiæ had been experienced. He could distinguish light from darknesss with the affected eye, but that was all. He was unable to give any account of the cause of the attack, but after a while he remembered that two months before losing his vision several teeth had been stopped. He had often felt pain near the first molar tooth of the right side, and he had often also experienced pain and tenderness of the gums, and abscesses had formed which he had himself opened with a penknife. On examination the tooth was found to be reduced to a shell, with a fistulous opening leading to the alveolus. Dr. de Witt, suspecting irritation of the fifth nerve proceeding from the teeth, removed the stopping in order to obtain a counter-opening, and to permit the fistulous orifice to close and to arrest the irritation. The immediate effect was that the gum ceased to be sore, and the visual power began to improve. After three weeks, when the sharpness of vision had greatly improved, the gums again began to be sore, and coincidently the acuteness of vision diminished. The affected tooth was extracted, and improvement then again took place, and vision became restored almost to its normal amount, though still not equal to that of the other. The fang of the tooth was filled with pus.

M. Nicase† remarks that he knew that in certain cases the irritation of a dental nerve may induce troubles, which

---

* "*Amer. Jour. Med. Science, N. S.*," CX. p. 382, April, 1868.

† "*Gaz. Med. de Paris*," 1871 p. 150.

indeed are often only transitory, of vision. These disturbances are explicable on the ground of the intimate relations which exist between the different branches of the fifth pair of nerves, the function of which is sensory, and which govern also the nutrition of the regions to which they are distributed. If the irritation of the fifth nerve is persistent, and if it ends by effecting some alteration in the nerves, it is possible that the disturbances of vision may become more serious and even permanent. Glaucoma, which is especially due to troubles of nutrition, may possibly have its point of departure in a lesion affecting the superior or inferior dental nerves.

The relations of the teeth to glaucoma have been considered by several writers, but especially by Hermann Schmidt* and by Priestly Smith, whose remarks upon the subject are very sensible.†

Glaucoma is a condition of the eye in which the humors of the eye are secreted in larger quantity than they can be taken up by the absorbents; the tension of the eye is therefore increased; it becomes hard and exquisitely tender, then extremely painful; the vessels of the retina are often seen to pulsate, and finally, after a period of suffering of variable duration, it quiets down, but the disk remains cupped, and vision is either altogether lost, or permanently and seriously impaired. I may say, in passing, that the cases of blindness which come before the ophthalmic surgeon from this cause are amongst the most melancholy that he sees—melancholy because he feels that in the majority of cases, if the surgeon in attendance, instead of being misled by the specious term "neuralgia," had recognized the disease and insisted on the performance of iridectomy during the first two or three days of pain, sight would have been perfectly preserved.

In regard to the pathology of this disease it is enough to state here that the increased hardness or tension of the eye

---

* *Archiv. f. Opthal,*" XIV, i., 107.

† "Glaucoma; Its Causes, &c.," 1879, p. 10 *et seq.*

is due in part to hpper-secretion, and in part to imperfect drainage to the aqueous fluid. How are these conditions produced? The imperfect drainage we know is produced by a narrowing of the depth of the anterior chamber, which interferes with the entrance of the aqueous humor into the canal of Schlemm and its passage outwards, and this is the result of the approximation of the iris to the cornea, either by enlargement of the lens, as Priestly Smith thinks, or by the formation of adhesions between the iris and cornea, as others believe.

But is there any evidence of hyper-secretion, and if there be, is the fifth nerve the exciting or even a predisposing cause?

It is certain that pain in the region of one or other of the branches of the fifth is a precursor of glaucoma in a large number of cases; and the presence or absence of ciliary neuralgia is mentioned in the report of almost every glaucomatous case.

But the most convincing evidence of the point in question is that which is derived from the experiments of Von Hippel and Grunhagen.* These observers found that whilst the normal tension of the contents of the eyeball is about one inch of mercury, if the aorta were compressed the tension rose to about three inches of mercury, and if now the fifth nerve were stimulated the pressure rose to as much as eight inches of mercury, and at the same time well-marked pulsations were observed. Even when the stimulation of the fifth was discontinued the tension was permanently increased, and the augmentation was perceptible even after death. It is not clear whether the trigeminus here acts as a true secreto-motory nerve in the same sense that stimulation of the chorda tympani causes increased activity of the salivary secretion, or whether it acts simply by causing dilatation of the blood vessels, and thus leading to an increased supply of blood to the organ.

---

* "*Archiv. f. Ophlhal.*," XIV., iii. pp. 219-258

The determination of this point, however, matters little from our present point of view. It is sufficient that irritation of the fifth causes marked increase in the tension of the eye, and it is easy to understand that in all cases where, from physical changes consequent on gouty or rheumatic inflammation of the iris, the eye is ripe for an outburst of glaucoma, the spark may be supplied by the irritation of a carious tooth.

It is but right to add, however, that Priestly Smith has made a series of tonometrical experiments at the Dental Hospital in a series of cases of toothache. The results he obtained were not very decisive, though he arrived at the conclusion that in young persons toothache rarely, if ever, causes a decided rise of tension. He estimated the tension in sixteen persons suffering at the moment or recently from severe pains in the teeth, and found on the whole little or no appreciable difference.

The wasting of this paper has recalled to my memory various cases which were in the highest degree suggestive of the teeth being the starting point of the trouble, and to which I regret extremely I did not direct a larger amount of care and attention at a period when it might have proved of service. To give one only :—

Mrs. S., æt. 32, a stout, ruddy-complexioned woman. suffered in 1878 and 1879 from colored haloes round lights, and the general symptoms of threatening glaucoma. For these there was do apparent cause. She was healthy, in easy circumstances, and her habit of body and general manner of life were thoroughly normal and good. She, however, suffered severely from toothache. A particular tooth would begin to ache, without apparently decay of the crown. After enduring the pain for some time she would have the tooth extracted, and an abscess was always found to exist at the root. In March, 1880, after a fall which intensified all the symptoms, an iridectomy was performed on the left eye. The wound did not heal kindly, and a cystoid cicatrix was the result. The vision gradually deteriorated, and ultimately was entirely lost.

She now had two miscarriages, and some circumstances occurred in the family causing her much mental distress and depression of spirits, with frequent shedding of tears. I saw her first about eighteen months after the operation had been done.

The right eye was now the seat of trouble, but the vision was still very good, and with appropriate glasses (+ 1 D) could be rendered perfect. Three weeks previously she had awaked with violent pain, which lasted for three days. She saw bright lights, and the eye was hard, and the optic disk was white and cupped. She was told she had glaucoma, and that if the vision deteriorated she must submit to an operation. It did deteriorate rapidly, and I performed an iridectomy in the hope of saving vision, but the wound passed through the same stages as with the previous operation, and a cystoid cicatrix was formed with persistent high tension of the globe of the eye. I saw her only a few days ago with complete blindness of the left eye, and she repeated to me the story of the teeth, and I could not but think that if a similar case appeared before me now, instead of trying iridectomy—which was here a signal failure, even when performed by two different operators, and which resisted eserine, and all the usual methods of reducing tension—I should at once have every tooth carefully and thoroughly inspected by an experienced dental surgeon. And I would ask whether such a case is not adapted for the removal and replantation of teeth, if these, after extraction, were found to be sound.

I, fear, Mr. President and Gentlemen, that I have wearied you with cases, but I trust I may stand excused when I say that most of these cases are germane to the matter, and that my object has been in part to draw the attention of some amongst you who may not have given it a thought, to the close relationship which exists between the teeth and the eye, different as these organs are in their mode of development, their histological characters, and physiological uses, and in part to elicit from you some information that may prove of service to the ophthalmic surgeon, and may afford him some hints of the kinds of disease of the teeth which he is likely to overlook, and which it is important that he should recognize.

In conclusion, then, I think it may be laid down as a maxim to be generally observed that in all cases of threatening glaucoma, especially when this is associated with ciliary neurosis and obscure pains in the temples and maxillary orbital regions—in all cases of mydriasis, and probably of myosis, originating without apparent causes—in all cases of sudden paralysis, of either the orbital muscles, or of loss of sensation in the absence of cerebral symptoms—in all cases of phlyctenular disease of the conjunctiva—in all cases of ulcers of the cornea resisting ordinary treatment—in all cases of sudden failure of accommodation, especially in young children—and finally, in cases of exophthalmia, the condition of the teeth should at least be examined, and if faulty conditions present themselves these should be at once rectified, and then one at least of the possible causes of each of these diseases will be removed.—*Dental Record*.

---

ARTICLE II.

